# Supplementary material for: Bridging the “know-do” gap to improve active case finding for tuberculosis in India: A qualitative exploration into national tuberculosis elimination program staffs’ perspectives
Source: PLoS One. 2024 Nov 4;19(11):e0309750. doi: 10.1371/journal.pone.0309750 (PMC11534205; doi:10.1371/journal.pone.0309750)
Supplement: Attachment — Submitted filename: PLOSone Review 19.3.2024.docx [file pone.0309750.s001.docx]

The study is from a commissioned TB ACF evaluation project by India’s National TB Elimination (NTEP). India is trying to explore implementation of ACF, however, considering the costs and comparative insignificant case finding, passive case finding remained the cornerstone of NTEP for decades. The present study brings out some experiences based on qualitative research which is an important aspect. It is also important that authors addressed “know-do gap” in the ACF implementation. However, in my opinion in the current form it has several limitations in terms of generalizability and overall analysis. Authors may need to undertake a detailed revision.

1. The title should reflect “NTEP” program otherwise just stating program does not give an idea to readers.
2. Abstract: Objective should not include suggested solutions
3. 34 key informant interviews should go as a part of methods.
4. Authors mentioned that they performed manual analysis, but in my opinion, it may sometimes be biased. Why did authors not use qualitative data management programs such as MAXQDA or Nvivo etc.? That would provide more objective analysis.
5. Lines 78-79: Authors mentioned “ACF did not have better outcome rates as compared to Passive case finding”. This needs to be interpreted carefully. Treatment outcomes do not necessarily always depend upon ACF and early diagnosis, but the entire treatment course as well. So, the comparison needs to be made considering various points in both methods. The rationale for undertaking qualitative research is not sufficiently stated.
6. The real problem with ACF implementation is whether incentives are going to continue as that would decide the sustainability of any program.
7. Methods section should be revised thoroughly as how actually they did the data management and analysis. Authors may describe how content analysis underpinned current research. Authors should describe the detailed method of thematic analysis.

Authors mentioned in the abstract that two investigators did a manual descriptive thematic analysis of the transcripts, independent of each other. A third investigator reviewed the same. It is unclear whether there were consistency or discrepancies in this method and how third person contributed. Authors may want to throw light on these issues.

8. Lines 218-220: “The data collected from project research assistants was about ACF implementation by NTEP and not about their own performance. Hence, we do not think that this relationship between investigator and project research assistants would have affected the richness of data”. This is not clear.
9. Ethics approval should be in the beginning of methods section.
10. In my opinion, some quantification from 34 interviews might help in some places to understand how relevant is that issue and its importance. Authors used “many of the” which is hard to follow.
11. The analysis needs to be organized. Authors mentioned numerous themes clubbed into a few categories. It is hard to follow.
12. The discussion should highlight that ACF strategies need to be implemented considering the ground situations which is often lacking in Indian context. Capacity building and quality improvement are very general suggestions as these are constant processes. In my opinion authors need to provide suggestions based on their own specific findings which will help to undertake focused interventions.
13. Lines 472-473: “In line with our findings, studies elsewhere have also reported similar

challenges [22–26]. Authors should first describe what other studies have reported and then discuss their findings whether they are confirming or having different observations.

14. The conclusion needs to be specific and based on findings rather than general.
