# Supplementary material for: Bridging the “know-do” gap to improve active case finding for tuberculosis in India: A qualitative exploration into national tuberculosis elimination program staffs’ perspectives
Source: PLoS One. 2024 Nov 4;19(11):e0309750. doi: 10.1371/journal.pone.0309750 (PMC11534205; doi:10.1371/journal.pone.0309750)
Supplement: Attachment — Submitted filename: Response to reviewers.docx [file pone.0309750.s002.docx]

**Title:** Bridging the “know-do” gap to improve active case finding for tuberculosis in India: A qualitative exploration into national tuberculosis elimination program staffs’ perspectives

**[Kindly note a change in title based on a comment by reviewer]**

**PONE-D-23-43822**

**Article type:** Research Article

**Journal**: PLOS One

**Sub: Submission of revised manuscript**

**Dear Editor,**

We would like to express our sincere gratitude for the constructive comments from the reviewers. We found them very helpful for improving the quality of our manuscript. We have made every effort to address each comment as carefully as we can. All revised parts in the manuscript are in track change. Please find our point-by-point responses below. **The line numbers mentioned in our response are the line numbers from the “Revised Manuscript with Track Changes”.**

We have also taken this opportunity to make some editorial changes for better ease of reading. If there are any further clarifications, we would be very glad to address them,

The correct updated funding statement is as follows

ICMR-NIE led USAID/JSI supported TB ACF evaluation project is a collaborative effort involving ICMR-National Institute of Epidemiology (ICMR-NIE), Chennai, India (lead); USAID India, New Delhi, India; JSI India, New Delhi, India; The WHO Country Office for India, New Delhi, India; and Central TB Division, Ministry of Health and Family Welfare, Government of India, New Delhi, India. This study was commissioned by India’s national TB elimination program, with funding support from USAID through John Snow International (JSI) under the TB Implementation Framework Agreement (TIFA). There was no additional external funding received for this study. The contents of this study document are the authors' sole responsibility and do not necessarily reflect the views of USAID or the United States Government.

With regards,

Dr Hemant D Shewade

**Responses to Comments**

*******************************************

**Journal Requirements:**

1. When submitting your revision, we need you to address these additional requirements.

Please ensure that your manuscript meets PLOS ONE's style requirements, including those for file naming. The PLOS ONE style templates can be found at

https://journals.plos.org/plosone/s/file?id=wjVg/PLOSOne_formatting_sample_main_body.pdf and

<https://journals.plos.org/plosone/s/file?id=ba62/PLOSOne_formatting_sample_title_authors_affiliations.pdf>

AUTHOR RESPONSE

Noted with thanks. We have ensured that manuscript meets PLOS ONE style requirements

**Journal Requirements:**

2. We note that the grant information you provided in the ‘Funding Information’ and ‘Financial Disclosure’ sections do not match.

When you resubmit, please ensure that you provide the correct grant numbers for the awards you received for your study in the ‘Funding Information’ section.

AUTHOR RESPONSE

We have removed funding information from manuscript file and ensured correct statement in the manuscript submission website and mentioned the same in the cover letter. We have removed funding related information any other parts of the manuscript.

**Journal Requirements:**

3. Thank you for stating in your Funding Statement:

This study was commissioned by India’s national TB elimination program, with funding support from USAID through John Snow International (JSI) under the TB Implementation Framework Agreement (TIFA). The contents of this study document are the authors' sole responsibility and do not necessarily reflect the views of USAID or the United States Government.

Please provide an amended statement that declares all the funding or sources of support (whether external or internal to your organization) received during this study, as detailed online in our guide for authors at http://journals.plos.org/plosone/s/submit-now. Please also include the statement “There was no additional external funding received for this study.” in your updated Funding Statement.

Please include your amended Funding Statement within your cover letter. We will change the online submission form on your behalf.

AUTHOR RESPONSE

We have removed funding information from manuscript file and ensured correct statement in the manuscript submission website and mentioned the same in the cover letter. We have removed funding related information any other parts of the manuscript.

**Journal Requirements:**

4. In the online submission form, you indicated that The anonymized transcripts are available on request to the corresponding author subject to signature of a data confidentiality agreement.

All PLOS journals now require all data underlying the findings described in their manuscript to be freely available to other researchers, either a. In a public repository, b. Within the manuscript itself, or c. Uploaded as supplementary information.

This policy applies to all data except where public deposition would breach compliance with the protocol approved by your research ethics board. If your data cannot be made publicly available for ethical or legal reasons (e.g., public availability would compromise patient privacy), please explain your reasons on resubmission and your exemption request will be escalated for approval.

AUTHOR RESPONSE

Data cannot be shared publicly. In this qualitative study, the transcripts are the data. The transcripts, even if anonymized, risk the confidentiality of the person who provided the information / perspective. In the patient information sheet and consent form that was signed, we do not have the consent to share the transcript from the interviews. Hence, sharing the transcript will breach the compliance with the protocol approved by the ICMR-NIE’s institute human ethics committee. Data are available from the ICMR-NIE’s institute human ethics committee (contact via corresponding author) for researchers who meet the criteria for access to confidential data. The anonymized transcripts are available on request to the corresponding author subject to signature of a data confidentiality agreement.

The same has been reflected in the data availability statement in the tail section of the manuscript.

**Journal Requirements:**

5. Thank you for stating the following in the Acknowledgments Section of your manuscript:

ICMR-NIE led USAID/JSI supported TB ACF evaluation project is a collaborative effort involving ICMR-National Institute of Epidemiology (ICMR-NIE), Chennai, India (lead); USAID India, New Delhi, India; JSI India, New Delhi, India; The WHO Country Office for India, New Delhi, India; and Central TB Division, Ministry of Health and Family Welfare, Government of India, New Delhi, India. The authors sincerely thank the support and contribution received from all the state TB cells, district TB cells and the WHO NTEP medical consultant network in India. We also acknowledge the contribution by the interns of ICMR NIE, Chennai, India who provided data transcription support: Mr SM Aakash, Ms M Nisha, Ms Archita Govardhana, Ms Suhana Khatoon B, Ms Aishwarya Dhumale and Mr Mahesh Gomasa.

We note that you have provided funding information that is not currently declared in your Funding Statement. However, funding information should not appear in the Acknowledgments section or other areas of your manuscript. We will only publish funding information present in the Funding Statement section of the online submission form.

Please remove any funding-related text from the manuscript and let us know how you would like to update your Funding Statement. Currently, your Funding Statement reads as follows:

This study was commissioned by India’s national TB elimination program, with funding support from USAID through John Snow International (JSI) under the TB Implementation Framework Agreement (TIFA). The contents of this study document are the authors' sole responsibility and do not necessarily reflect the views of USAID or the United States Government.

Please include your amended statements within your cover letter; we will change the online submission form on your behalf

AUTHOR RESPONSE

We have removed funding information from manuscript file and ensured correct statement in the manuscript submission website and mentioned the same in the cover letter. We have removed funding related information any other parts of the manuscript.

The correct updated funding statement is as follows

ICMR-NIE led USAID/JSI supported TB ACF evaluation project is a collaborative effort involving ICMR-National Institute of Epidemiology (ICMR-NIE), Chennai, India (lead); USAID India, New Delhi, India; JSI India, New Delhi, India; The WHO Country Office for India, New Delhi, India; and Central TB Division, Ministry of Health and Family Welfare, Government of India, New Delhi, India. This study was commissioned by India’s national TB elimination program, with funding support from USAID through John Snow International (JSI) under the TB Implementation Framework Agreement (TIFA). There was no additional external funding received for this study. The contents of this study document are the authors' sole responsibility and do not necessarily reflect the views of USAID or the United States Government.

*******************************************

**Reviewer #1 (Please note: The line numbers mentioned in our response are the line numbers from the “Revised Manuscript with Track Changes”)**

**REVIEWER**

The manuscript, “Bridging the “know-do” gap to improve active case finding for tuberculosis in India: A qualitative exploration into program staffs’ perspectives” describes the enablers, barriers, and suggested solutions to improve TB active case finding India from NTEP staff (provider) perspective.

The authors have described well the introduction, and results. The methodology and the discussion need some improvement. The findings have public health importance to understand what factors may influence TB active case findings. The findings may also have importance in the context of infection prevention and control in community settings. However, the manuscript would require substantial revision to make it clearer and reader friendly.

The manuscript may be appropriate for this journal and could be considered for publication in PLOS ONE after addressing following issues.

**AUTHORS’ RESPONSE**

Thank you very much for the comment. We appreciate your constructive comments.

**REVIEWER**

Abstract:
The objective could be merged with the background section for clarity and conciseness

**AUTHORS’ RESPONSE**

Thank you very much for the suggestion. The objective has been merged with the background as suggested. (line 25-32 of revised manuscript with track changes)

**REVIEWER**

Abstract Methods:
Additional details are required in this section. The analysis section is very vague and warrants more information. This includes clarification on whether the interviews were recorded, how key informants were selected and why, the type of questions asked, who transcribed the recordings, whether the transcriptions were verbatim, how themes were identified, and if there were any pre-defined themes. Additionally, the resolution of inconsistencies between the two individuals involved in thematic analysis should be explained.

**AUTHORS’ RESPONSE**

Thank you very much for the comment. These mentioned additional details have been incorporated into the methods section of the abstract. (line 35-44 of revised manuscript with track changes)

**REVIEWER**

Abstract Results:
Clarify the term "Ni-kshay" as an international audience may not understand it. The conclusion regarding revising ACF guidance is too generic. Authors should specify the changes required based on the study findings

**AUTHORS’ RESPONSE**

Thank you very much for the comment. The term “Ni-kshay” has been clarified in the result section of the abstract. (line 55-56 of revised manuscript with track changes)

Additionally, we have clarified the revision required in ACF guidance in the conclusion section of the abstract. (line 60-65 of revised manuscript with track changes)

**REVIEWER**

Main manuscript-
Introduction- The statement “Based on the data available, we used three derived ACF quality indicators in our analysis (see Table 1) [16]. We observed that comprehensive mapping of the high-risk populations in the districts was not done at the beginning of the year.” sounds more like a description of the study methods and should be revised accordingly.

**AUTHORS’ RESPONSE**

Thank you for your comment.

We would like to clarify that this national-level evaluation of TB-ACF was conducted in a phase-wise manner.

Under the first phase (secondary data analysis) of this evaluation, we assessed the frequency, scale and quality of ACF at the district, state and national level for the year 2021 using routinely collected secondary ACF data in *Ni-kshay* (TB information management system under NTEP). The results have been published in the Global Health Action journal and the reference has been provided.

Due to data related issues, we were not able to calculate all the ACF quality indicators. In the main text, we have clarified how we generated the revised indicators (using the data available) and interpreted the results of the first phase, based on which broad open-ended questions were developed for this descriptive qualitative study. Hence, we find it apt to provide clarification in the introduction section of the main text.

The study method used for descriptive qualitative study has been provided in the methods section.

**REVIEWER**

Study settings- Estimations regarding the number of sub-district (block) level administrative TB units and peripheral health institutions (public and private) providing TB diagnosis and treatment services should be provided. The study location, including the names of the districts and reasons for their selection, should be mentioned. Avoid unnecessary information.

**AUTHORS’ RESPONSE**

Thank you for your comment.

We have now provided the number of sub-district (block) level administrative TB units and peripheral health institutions (public and private) providing TB diagnosis and treatment services. (line 143-48 of revised manuscript with track changes)

The names of districts and reason for their selection (purposive) has been mentioned under the sub-section “study population”.

Other details in the study setting in the current form are required for an international audience to understand what happens under ACF routinely within the NTEP. We have also provided information on TB ACF guidelines released by the Central TB Division in India. We also refer to these points clarified in the settings, later in the methods, results and discussion. Hence, we find this is apt to be included in the study setting.

Considering the flow of the write up, the author group has decided to retain sampling strategy, purposive selection of districts along with reason for selection in the study population sub-section. Reason has been provided below. Study population includes description by time place and person. Hence, the place is part of the study population (the district/state to which the study participants belong). Additionally, we followed purposive sampling strategy to include these districts. The purpose for including these districts has also been mentioned in the study population sub-section. As sampling is part of study population subsection, we decided to retain this aspect (sampling strategy and district included) under study population. We hope this is fine.

**REVIEWER** Study population- Focus solely on describing the study population in this section.
“These 30 NTEP districts from nine states formed a convenient, accessible sample for the qualitative systematic enquiry (second phase of the project). Among these 30, six districts (from six different states) were purposively chosen: Ahmedabad Municipal Corporation (Gujarat), Jaipur I (Rajasthan), Aurangabad (Bihar), East Khasi Hills (Meghalaya), Deogarh 167 (Odisha), Pudukkottai (Tamil Nadu) (see Fig 2)” these should go under the study settings.

**AUTHORS’ RESPONSE**

Thank you for your suggestion. This is a good idea which we considered. However considering the flow of the write up, the author group has decided to retain this in the study population sub-section. Study population includes description by time place and person. Hence, the place is part of the study population (the district/state to which the study participants belong). Additionally, we followed purposive sampling strategy to include these districts. The purpose for including these districts has also been mentioned in the study population sub-section. As sampling is part of study population subsection, we decided to retain this aspect (sampling strategy and district included) under study population. We hope this is fine.

**REVIEWER**

Data collection- Clarify how consent was obtained for the second round of interviews and whether it was planned in the study protocol. Explain the steps taken to maintain confidentiality and mention any refusals for the second round of interviews. Additionally, state the number of repeated interviews conducted.

**AUTHORS’ RESPONSE**

Thank you for your comment. In the protocol, we had mentioned that if required, repeat interviews would be conducted and consent for this was taken during the initial round of consent itself. Total number of interviews and number of repeat interviews have been mentioned in the result section.

**REVIEWER**

Data management and analysis- Provide details regarding the number of people involved in data transcription and the average length of interviews. In Table 2, ensure the last column is labelled "Duration of interview in minutes."

**AUTHORS’ RESPONSE**

Thank you for your comment and suggestion. The mentioned points have been included in the data management and analyses section. (line 239-56 of revised manuscript with track changes) In Table 2, the last column has been labelled as you mentioned. Regarding the average length of interviews, this information has been included in the results section.

**REVIEWER**

Discussion- When referring to similar studies, mention the location to provide context for the audience. Interpret the findings rather than just describing them. Explain why knowledge gaps exist and provide reasoning for identified discrepancies.

**AUTHORS’ RESPONSE**

Thank you for your comment. The location has been mentioned to provide context for the audience (see 496-507). The reasons for the existence of knowledge gaps and identified discrepancies have been provided. (line 533-534, line 528-39 of revised manuscript with track changes)

*****************

**Reviewer #2 (Please note: The line numbers mentioned in our response are the line numbers from the “Revised Manuscript with Track Changes”)**

**REVIEWER**

The study is from a commissioned TB ACF evaluation project by India’s National TB Elimination (NTEP). India is trying to explore implementation of ACF, however, considering the costs and comparative insignificant case finding, passive case finding remained the cornerstone of NTEP for decades. The present study brings out some experiences based on qualitative research which is an important aspect. It is also important that authors addressed “know-do gap” in the ACF implementation.

**AUTHORS’ RESPONSE**

Thank you very much for the comment. We appreciate your constructive comments.

**REVIEWER**

The title should reflect “NTEP” program otherwise just stating program does not give an idea to readers.

**AUTHORS’ RESPONSE**

Thank you for your comment. Based on the feedback, the revised title is as follows.

**Bridging the “know-do” gap to improve active case finding for tuberculosis in India: A qualitative exploration into national tuberculosis elimination program staffs’ perspectives**

**REVIEWER**

Abstract: Objective should not include suggested solutions

**AUTHORS’ RESPONSE**

Thank you for your comments. We would like to clarify that our objectives were,

a. To understand the enablers and barriers to TB ACF implementation in India from providers' perspective

and

b. To also explore the suggested solutions in addressing the barriers identified from the providers' perspective.

Hence, we have included suggested solutions in our objective section. These are suggested solutions from NTEP staff perspective. In qualitative studies (conducted in the context of OR/IR or program evaluation), it is common practice to explore the suggested solutions from provider perspective to address the challenges.

In the discussion, we have clarified that based on the identified barriers and suggested solution (as perceived by the NTEP staff) we inferred a ‘know-do’ gap. Then we presented our recommendations (author recommendations). These recommendations were based on the suggested solutions (as perceived by NTEP staff) and authors’ experience in NTEP. Please see lines 509 -12 and 558-60 in the revised manuscript with track changes where these points have been clarified.

**REVIEWER**

**Abstract.** 34 key informant interviews should go as a part of methods.

**AUTHORS’ RESPONSE**

Thank you for your comments. This is a qualitative study. Since our sample size was not predetermined and was guided by data saturation, we were able to access the number of participants interviewed only at the end of the second round and repeated interviews. Hence, we have included 34 key informant interviews under the results section*.* We have maintained this consistency in reporting numbers in results section: in abstract as well as main text.

**REVIEWER**
Authors mentioned that they performed manual analysis, but in my opinion, it may sometimes be biased. Why did authors not use qualitative data management programs such as MAXQDA or Nvivo etc.? That would provide more objective analysis.

**AUTHORS’ RESPONSE**

Thank you for your comment and query.

Thinking is the core of coding, categorization and formation of themes. We feel that computer software only assists in the process of analysis. Considering the researcher's comfort, project requirements and less number of interviews, the choice of software was not pre-decided during the proposal development and we have not planned to get full versions of software. Hence, we used conventional “cut and paste” methods and used colours to code the text information in MS Word document. Additionally, the researchers have also previously conducted small-scale studies and have used manual thematic analysis to get insight into data analysis.

Hence, two independently trained researchers in qualitative research undertook manual descriptive thematic analysis and analysed the transcripts. A third experienced investigator reviewed it to reduce bias and increase interpretive credibility. The third investigator also resolved any inconsistencies in the same. The final results after analysis were shared with the stakeholders for their feedback and approval.

We hope this is fine.

**REVIEWER**
Lines 78-79: Authors mentioned “ACF did not have better outcome rates as compared to Passive case finding”. This needs to be interpreted carefully. Treatment outcomes do not necessarily always depend upon ACF and early diagnosis, but the entire treatment course as well. So, the comparison needs to be made considering various points in both methods. The rationale for undertaking qualitative research is not sufficiently stated.

**AUTHORS’ RESPONSE**

Thank you for the comment.

We beg to differ with the reviewer comment here. One of the expected benefits of ACF is better treatment outcomes (as per WHO document). Whenever comparison of outcomes is done among ACF and PCF detected patients, it is done after adjusting for baseline confounders. The same was done in the paper cited by the authors, in other words comparison was made after considering other predictors of treatment outcomes in the paper cited. We have mentioned the same in the introduction.

Regarding the rationale for qualitative research, based on the feedback, we have provided more clarify in the last paragraph of introduction section. Please see lines 117-122 of revised manuscript with track changes.

**REVIEWER**
The real problem with ACF implementation is whether incentives are going to continue as that would decide the sustainability of any program.

**AUTHORS’ RESPONSE**

Thank you for your comment. We agree with the reviewer that providing incentives would decide the sustainability of any program. Yes, the incentives are going to be continued in NTEP. The states may individually develop guidelines for TB ACF incentives based on their context.

As we have mentioned in the results, despite the existence of incentives, the coverage of disbursement remains suboptimal as perceived by NTEP staff and ACF yield remains low. Lack of timely incentives may lead to compromise in the quality of ACF and staff become less motivated to conduct ACF.

We have also recommended quality control linked disbursement of incentives. As we have suggested in our recommendations, incentives should be granted to field staff or volunteers for each household visited and for every specimen collection and transport to the laboratory. Over a pre-decided period (required for sufficient sample size to assess test positivity), specific quality control criteria need to be fulfilled before the release of these incentives.

We have discussed incentives, Please see line 324-34, 369-76, 471, 484-89, 528-39, 566-80 of revised manuscript with track changes

**REVIEWER**
Methods section should be revised thoroughly as how actually they did the data management and analysis. Authors may describe how content analysis underpinned current research. Authors should describe the detailed method of thematic analysis.
Authors mentioned in the abstract that two investigators did a manual descriptive thematic analysis of the transcripts, independent of each other. A third investigator reviewed the same. It is unclear whether there were consistency or discrepancies in this method and how third person contributed. Authors may want to throw light on these issues.

**AUTHORS’ RESPONSE**

Thank you for the comment. Regarding how content analysis underpinned current research, we used inductive reasoning to condense the raw narrative data into themes and categories based on valid inference and interpretation. We have clarified this and the additional requested details in the data collection and analysis section. Please see 207-56 lines of revised manuscript with track changes

**REVIEWER**

Lines 218-220: “The data collected from project research assistants was about ACF implementation by NTEP and not about their own performance. Hence, we do not think that this relationship between investigator and project research assistants would have affected the richness of data”. This is not clear.

**AUTHORS’ RESPONSE**

Thank you for your query.

Please note that this study reports the findings of Phase II of the TB ACF evaluation project. Under this phase II (qualitative systematic enquiry), we explore the why and how of the findings of the first phase (national level ACF secondary data analysis into ACF quality). Phase III was conducted in 30 randomly sampled districts to prospectively capture ACF care cascade data to generate reliable ACF quality indicators, know the extent of use of rapid molecular tests in ACF care cascade and delays in ACF care cascade. Additionally in phase III, we aimed to understand the differences in pre-treatment delays among ACF- and PCF-detected patients. In the first part of Phase III (prospectively generated ACF care cascade data), the district project research assistant (one per district) ensure that in the 30 randomly sampled district, the ACF tools is used in the field to capture individual level data of all those screened. He/she coordinated the same and compiled all the ACF tools used during ACF days in the 2023 in his / her district. He also conducted patient interviews for pre-treatment delay under the next part of phase III. To summarise, our project research assistants were simultaneously posted in 30 districts of 9 states in India and they prospectively evaluated TB ACF implementation at the field level (Phase 3) using the paper-based tool. They remained in constant touch with the investigators from the beginning of the study. The investigators felt that as they worked in close coordination with NTEP staff in the district for over an year and coordinate phase III of ACF evaluation project, they could provide rich information into barriers of ACF implementation.

As per the COREQ checklist, we had to report if the relationship with the participants was established before the study commencement.

We have purposively selected five research assistants for this key informant interviews under phase II. The previously established relationship between the investigator and project research assistants did not affect the richness of data as the questions were related to ACF implementation by NTEP and not about their performance in collecting data under phase III.

We hope this is now clear.

**REVIEWER**
Ethics approval should be in the beginning of methods section.

**AUTHORS’ RESPONSE**

Thank you for the comment. After looking at the location of the ethics statement in other studies published in PLOS One, we authors reached a consensus to retain the ethics approval statement at the end of the methods section. I hope this is fine.

**REVIEWER**
In my opinion, some quantification from 34 interviews might help in some places to understand how relevant is that issue and its importance. Authors used “many of the” which is hard to follow.

**AUTHORS’ RESPONSE**

Thank you for the comment.

After attaining consensus among the co-authors authors, we have decided to refrain from quantifying 34 interviews. We feel that even a category/theme that is only mentioned by a small number of respondents can be a valuable contribution to our data analysis which we may lose by quantification. Additionally, the purpose of qualitative studies is not to quantify but to explore the why and how (enablers, barriers, and suggested solutions).

**REVIEWER**
The analysis needs to be organized. Authors mentioned numerous themes clubbed into a few categories. It is hard to follow.

**AUTHORS’ RESPONSE**

Thanks for the comment. One an average there are not more than 3-4 themes clubbed under one category. We have also used figures to summarize the themes and categories (see Fig 3 and 4)

Under enablers, we identified six themes which were broadly classified into two major categories, ‘operational planning and stakeholder engagement’ and ‘technological advancements’ (Fig 3)

Under barriers, we identified 11 themes broadly classified into four major categories, ‘administrative’, ‘logistic’, ‘technical’ and ‘socio-cultural and geographic’. Under suggested solutions, we identified seven themes, broadly classified under two categories, ‘ACF quality improvement strategies’ and ‘capacity building strategies’. Fig 4 summarizes the barriers along with the relevant suggested solutions.

We hope this is fine

**REVIEWER**
The discussion should highlight that ACF strategies need to be implemented considering the ground situations which is often lacking in Indian context. Capacity building and quality improvement are very general suggestions as these are constant processes. In my opinion authors need to provide suggestions based on their own specific findings which will help to undertake focused interventions.

**AUTHORS’ RESPONSE**

Thank you for your comment.

We would like to clarify that our objectives were,

a. To understand the enablers and barriers to TB ACF implementation in India from providers' perspective

and

b. To also explore the suggested solutions in addressing the barriers identified from the providers' perspective.

Capacity building and quality improvement strategies were the categories that emerged from the suggestions (2^nd^ objective) provided by NTEP stakeholders.

Discussion section involves, discussing the suggested solutions by the providers / NTEP stakeholders. In addition, the authors also made an inference regarding the know-do gap that existed and made their own recommendations to improve ACF implementation. We have considered the ground situation.

Based on the suggested solutions by NTEP stakeholders, our inference of qualitative findings and authors’ own program experience, we have suggested six recommendations in the discussion section. These are very specific actionable recommendations. Please see lines 558-608 in the revised manuscript with track changes.

**REVIEWER**

Lines 472-473: “In line with our findings, studies elsewhere have also reported similar
challenges [22–26]. Authors should first describe what other studies have reported and then discuss their findings whether they are confirming or having different observations.

**AUTHORS’ RESPONSE**

Thank you for the comment. We have revised this paragraph as advised

**REVIEWER**

The conclusion needs to be specific and based on findings rather than general

**AUTHORS’ RESPONSE**

Thank you for the comment. The conclusion has been made specific based on our study findings. (line 620-32)

******************************

**GENERAL COMMENT TO BOTH REVIEWERS AND EDITOR**

We have added some recent references in the revised manuscript

Singh AA, Creswell J, Bhatia V. Framework for planning and monitoring active TB case finding interventions to meet the global targets in the COVID-19 era and beyond: South-East Asia perspective. PLOS Glob public Heal. 2021;1: e0000073. doi:10.1371/journal.pgph.0000073

Taylor M, Medley N, van Wyk SS, Oliver S. Community views on active case finding for tuberculosis in low- and middle-income countries: a qualitative evidence synthesis. Cochrane database Syst Rev. 2024;3: CD014756. doi:10.1002/14651858.CD014756.pub2

Hamilton K, Tolfree R, Mytton J. A systematic review of active case-finding strategies for tuberculosis in homeless populations. Int J Tuberc Lung Dis. 2018;22: 1135–1144. doi:10.5588/ijtld.17.0784
