# Supplementary material for: Bridging the “know-do” gap to improve active case finding for tuberculosis in India: A qualitative exploration into national tuberculosis elimination program staffs’ perspectives
Source: PLoS One. 2024 Nov 4;19(11):e0309750. doi: 10.1371/journal.pone.0309750 (PMC11534205; doi:10.1371/journal.pone.0309750)
Supplement: Attachment — Submitted filename: Response to comments.docx [file pone.0309750.s003.docx]

**Editor Comments**

GENERAL RESPONSE BY AUTHORS

The indicators in table 1 and Fig 1 include both indicators for ACF scale as well as ACF quality. We have used this opportunity to clarify this in the narrative of introduction and in title / body of Figure 1 and Table 1. Table 1 refers to the derived indicators used in our previously published secondary data analysis of ACF (year 2021) – this was because we did not have all the aggregate numbers (as depicted in Fig 1) to calculate all the indicators.

ACF scale means, within an ACF cycle, what proportion of mapped high-risk population (it should be adequately mapped) was screened. Of those screened, how many were diagnosed (NNS) is the indicator for ACF quality. If NNS is high, then one may look at the specific ACF quality indicators like – proportion of presumptive among screened, proportion of tested among presumptive and proportion diagnosed among tested. Please refer to Fig 1 for all the indicator of ACF scale and quality.

We have also used this opportunity to correct some spelling mistakes here and there.

Please note that Figure 3 has been revised based on reviewer comments.

COMMENT
1. Clarify the term "Ni-kshay" in the abstract as an international audience may not understand it
RESPONSE

We have clarified on first use in both abstract and narrative text. Please see lines 44 and 93 of revised manuscript with track changes. *Ni-kshay* is the electronic TB information management system under NTEP

COMMENT
2. Specify the changes required in ACF guidance based on the study findings in the abstract conclusion
RESPONSE

In abstract conclusion, we have already specified the changes required in ACF guidance which are as follows: emphasize capacity building, need to carry out ACF in high-risk (not general) population, quality control-linked incentives, and regular implementation monitoring of the activities.

COMMENT
3. Revise the statement about using derived ACF quality indicators in the introduction, as it sounds more like a description of the study methods
RESPONSE

This study plans to go in depth into the findings of our national level assessment of quality of ACF using aggregate secondary data for the year 2021. The section the editor is referring to refer to this previous study that has been cited as reference 16. The derived ACF quality indicators are also with reference to the reference 16.

However, to address reviewer comment we have revised the statement (changed from active to passive voice). We hope it is now fine.

COMMENT
4. Consider quantifying some findings from the 34 interviews to help understand the relevance and importance of issues
RESPONSE

We thank the editor for the comment. This being a qualitative study, the aim is to not quantify but to explore in detail the reasons for suboptimal quality (from provider perspective) and suggested solutions to improve (from provider perspective). As shared in lines 249-71 of revised manuscript with track changes (subsection data management and analysis), we carried out manual descriptive thematic analysis. However content analysis, on the other hand, involves **quantifying** data by counting the frequency of specific terms or concepts and organizing them. Our aim was not to quantify but explore the reasons for suboptimal ACF quality that was summarized as themes. We sincerely hope this is fine.

COMMENT
5. Organize the analysis better, as numerous themes are clubbed into a few categories making it hard to follow

RESPONSE

We thank the editor for the comment. Among enablers, we have six themes under two categories. We have now classified them under three categories. During a review based on your comment, we felt it would be right to divide the category ‘operational planning and stakeholder engagement’ into two separate categories. We have made edits to reflect this in revised figure 3 and the narrative text (see lines 297-363 of revised manuscript with track changes).

We reviewed the categories and themes under perceived barriers and think they are fine in the current form.

We reviewed the categories and themes under suggested solutions (as perceived by NTEP staff) and made some minor edits (lines 470-540 of revised manuscript with track changes) in the classification of themes under categories.

We have made edits here and there in the results narrative to improve clarity.

COMMENT
6. Highlight in the discussion that ACF strategies need to be implemented based on the local context
RESPONSE

We thank the editor for the comment. We have clarified this in lines 618-19 and 668-70 of revised manuscript with track changes (discussion section).

COMMENT
7. Provide details on how consent was obtained for the second round of interviews and whether it was planned in the protocol
RESPONSE

Yes. Repeat interviews (second round of interviews) were planned in the protocol. If required, we had proposed to conduct repeat interviews (second round of interviews) in the protocol. The patient information sheet that was provided during written informed consent (during first interview) mentioned that the participants may be contacted for repeat interviews. Hence, there was no need for consent for repeat interviews.

COMMENT
8. Explain the steps taken to maintain confidentiality and mention any refusals for the second round of interviews

RESPONSE

We have explained the steps in maintain confidentiality in lines 133-35 of revised manuscript with track changes (under ethics). Additionally steps were taken to ensure confidentiality while reporting the results as well.

There were no refusals to participate (initial or repeat interviews). This has been clarified in lines 211 and 235 of revised manuscript with track changes.

COMMENT
9. State the number of repeated interviews conducted in the results

RESPONSE

We have included this information in the first line of results narrative and in the footnotes of table 2. Please refer to revised manuscript with track changes.

COMMENT
10. Provide the average length of interviews in the results section

RESPONSE

We have included this information in the first para of results narrative and in table 2. Please refer to revised manuscript with track changes.


**Reviewer 2:**

GENERAL RESPONSE BY AUTHORS

The indicators in table 1 and Fig 1 include both indicators for ACF scale as well as ACF quality. We have used this opportunity to clarify this in the narrative of introduction and in title / body of Figure 1 and Table 1.

ACF scale means, within an ACF cycle, what proportion of mapped high risk population (it should be adequately mapped) was screened. Of those screened, how many were diagnosed (NNS) is the indicator for ACF quality. If NNS is high, then one may look at the specific ACF quality indicators like – proportion of presumptive among screened, proportion of tested among presumptive and proportion diagnosed among tested.

We have also used this opportunity to correct some spelling mistakes here and there.

Please note that Figure 3 has been revised based on reviewer comments.

COMMENT
Authors have addressed all the comments, however the manuscript requires considerable editing to get in a publishable form and see the flow. please see the attachement

RESPONSE

We thank the reviewer for the comment. Regarding your comments in the attachment, we have addressed them, point by point, below

COMMENT
The study is from a commissioned TB ACF evaluation project by India’s National TB Elimination (NTEP). India is trying to explore implementation of ACF, however, considering the costs and comparative insignificant case finding, passive case finding remained the cornerstone of NTEP for decades. The present study brings out some experiences based on qualitative research which is an important aspect. It is also important that authors addressed “know-do gap” in the ACF implementation. However, in my opinion in the current form it has several limitations in terms of generalizability and overall analysis. Authors may need to undertake a detailed revision

RESPONSE

We have noted this. We have addressed your comments, point by point, below

COMMENT
The title should reflect “NTEP” program otherwise just stating program does not give an idea to readers.

RESPONSE

We thank the reviewer for the comment. NTEP stands for national tuberculosis elimination program. The title already has the phrase ‘national tuberculosis elimination program’.

We had received this comment during round one of review and in line with this we had changed the title during the first round of review. We are intrigued. Has the reviewer viewed the original submission files (did not have national tuberculosis elimination program in the title) and not the revised version (round one) that had national tuberculosis elimination program in the title?

COMMENT
Abstract: Objective should not include suggested solutions

RESPONSE

We thank the reviewer for the comment. However, we beg to differ here. The objectives of this study were to explore the reasons for suboptimal ACF quality and suggested solutions, both from NTEP staff perspective. Hence, we have retained this as the objective. While we have analyzed and presented the findings in results (that includes suggested solutions from NTEP staff-provider perspective), we have also provided our recommendations in the discussion (see lines 618-62 of revised manuscript with track changes).

COMMENT
34 key informant interviews should go as a part of methods.

RESPONSE

We thank the reviewer for the comment. However, we beg to differ here. The number of interviews are guided by saturation and at the time of implementing the study or planning this study (what we did? – Methods), we were not sure of the exact numbers. Hence, this should go in the results section. This is more of a style issue. I have seen many authors mention the exact number of interviews in the methods itself. However, the author group, for reasons mentioned above, have provided the exact number of interviews along with repeat of interviews in the results section. We hope this is fine.

COMMENT
Authors mentioned that they performed manual analysis, but in my opinion, it may sometimes be biased. Why did authors not use qualitative data management programs such as MAXQDA or Nvivo etc.? That would provide more objective analysis.

RESPONSE

We thank the reviewer for the comment. Usually softwares are recommended when one needs to analyse a lot of data. Considering the number of interviews (approx. 30-40), we performed the analysis manually. The number of interviews were not huge enough to mandate the use of qualitative data analysis softwares and we are comfortable with manual analysis. MS Word with comments function on the right, with highlighting was used to identify codes and apt quotes. These were later combined into themes and categories. This was done independently by two authors and then a third author was involved, if required, to review the results and sort out discrepancies in codes and themes. The same has been clarified in lines 249-71 of revised manuscript with track changes (data management and analysis subsection). To be transparent, we have reported all points under data analysis section (following COREQ).

COMMENT
Lines 78-79: Authors mentioned “ACF did not have better outcome rates as compared to Passive case finding”. This needs to be interpreted carefully. Treatment outcomes do not necessarily always depend upon ACF and early diagnosis, but the entire treatment course as well. So, the comparison needs to be made considering various points in both methods. The rationale for undertaking qualitative research is not sufficiently stated.

RESPONSE

The line refers to inference drawn from a previous study conducted in a project setting in India (project Axshya) and this is not a finding of this study. The previous study is reference number 11.I am reproducing the para below

“During 2013-15, under The Global Fund-supported project *Axshya* (*Axshya* means without TB in Sanskrit) in India, ACF among high-risk populations improved case notification rates (CNRs) at the TB unit level compared to TB units implementing PCF (passive case finding) only [8]. During 2016-17, under the same project, ACF reduced health-system level diagnosis delay at the national level. ACF-detected patients incurred lower costs and lower chances of catastrophic costs during TB diagnosis than PCF-detected patients. ACF-detected patients did not have significantly better treatment outcomes when compared to PCF-detected patients [9–11]. One of the limitations was the lack of qualitative systematic enquiry to explore the provider and beneficiary perspectives for the above findings [12].”

COMMENT
The real problem with ACF implementation is whether incentives are going to continue as that would decide the sustainability of any program.

RESPONSE

We agree with the comment. Incentives should continue but they should be quality control linked. We have clarified this in detail in the discussion section (see lines 623-37 of revised manuscript with track changes) and have a separate figure for this as well as to what steps to follow to ensure quality control linked incentives (also see Fig 5).

COMMENT
Methods section should be revised thoroughly as how actually they did the data management and analysis. Authors may describe how content analysis underpinned current research. Authors should describe the detailed method of thematic analysis.

RESPONSE

We thank the reviewer for the comment. To be transparent, we have reported all points under data collection and data analysis section (following COREQ). For data collection, please refer to lines 218-47 of revised manuscript and for data management and analysis, please refer to lines 249-71 of revised manuscript with track changes.

For further clarity, as requested by the reviewer, we have added appropriate references under the data management and analysis section (see ref 24 and 25 in revised manuscript). In programmatic settings where the researchers want to explore the reasons for a certain problem, thematic analysis is the most appropriate. Hence, content analysis is the theoretical underpinning. Thematic analysis is appropriate for determining solutions to real world problems and we have clarified it in lines 249-71 of revised manuscript with track changes.

There are five theoretical underpinnings: content analysis (to systematically organize data into a structured format)., grounded theory, phenomenology, ethnography, and discourse analysis. Within the theoretical framework of ‘content analysis’, as a methods of data analysis, we decided to perform thematic analysis, not content analysis. Our interest was to explore themes around reasons for poor ACF quality and suggested solutions to improve it, both from NTEP manager and implementers’ perspective. We were not interested in quantifying the reasons, and this was not our aim.

COMMENT
Authors mentioned in the abstract that two investigators did a manual descriptive thematic analysis of the transcripts, independent of each other. A third investigator reviewed the same. It is unclear whether there were consistency or discrepancies in this method and how third person contributed. Authors may want to throw light on these issues.

RESPONSE

We thank the reviewer for highlighting this point. The third person reviewed the data if there were discrepancies in the codes generated and we then finalized the codes and themes based on mutual discussion and consensus. A third experienced investigator reviewed the analysis to reduce subjective bias and increase interpretive credibility. The third investigator also discussed and resolved any inconsistencies in the codes and themes generated among the two investigators. We have clarified this in lines 249-71 of revised manuscript with track changes.

COMMENT
Lines 218-220: “The data collected from project research assistants was about ACF implementation by NTEP and not about their own performance. Hence, we do not think that this relationship between investigator and project research assistants would have affected the richness of data”. This is not clear.

RESPONSE

In COREQ guidelines, it is recommended that the relationship between interviewer and interviewee be clarified. Here the interviewer was the project consultant who reviewed the project work of the research assistant (immediate reporting officer). However, the data collected was about routine ACF implementation, not about the project research assistant’s performance. Hence, we not think a bias would have been introduced despite the project research assistant being interviewed by their immediate reporting officer. To clarify this, the statement was added in the methods section. We are reproducing the paragraph below for clarity

“The investigators involved in data collection work in a national public medical research institute and are well-versed with the NTEP. The investigator who conducted the interviews (PR) is not part of NTEP (external member). She was responsible for overall management of the TB ACF evaluation project that included human resource management (includes project research assistants). The qualitative data collected from project research assistants was about ACF implementation by NTEP and not about their own performance (regarding phase three project related data collection). Hence, we do not think that this relationship between investigator and project research assistants would have affected the richness of data. ”

COMMENT
Ethics approval should be in the beginning of methods section.

RESPONSE

We have placed it at the beginning of methods section as stated by the reviewer.

COMMENT
In my opinion, some quantification from 34 interviews might help in some places to understand how relevant is that issue and its importance. Authors used “many of the” which is hard to follow.

RESPONSE

We thank the reviewer for the comment.. This being a qualitative study, the aim is to not quantify but to explore in detail the reasons for suboptimal quality (from provider perspective) and suggested solutions to improve (from provider perspective). As shared in lines 249-71 of revised manuscript with track changes (subsection data management and analysis), we carried out manual descriptive thematic analysis. While content analysis, on the other hand, involves **quantifying** data by counting the frequency of specific terms or concepts and organizing them. Our aim was not to quantify but explore the reasons (through thematic analysis) for suboptimal ACF quality that was summarized as themes.

To further clarify, there are five theoretical underpinnings: content analysis (to systematically organize data into a structured format)., grounded theory, phenomenology, ethnography, and discourse analysis. Within the theoretical framework of ‘content analysis’, as a method of data analysis, we decided to perform thematic analysis, not content analysis. Our interest was to explore themes around reasons for poor ACF quality and suggested solutions to improve it, both from NTEP manager and implementers’ perspective. We were not interested in quantifying the reasons, and this was not our aim.

We sincerely hope this is fine and is now clear.

COMMENT
The analysis needs to be organized. Authors mentioned numerous themes clubbed into a few categories. It is hard to follow.

RESPONSE

We thank the reviewer for the comment. We have used this opportunity to improve clarity.

Among enablers, we have six themes under two categories. We have now classified them under three categories. During a review based on your comment, we felt it would be right to divide the category ‘operational planning and stakeholder engagement’ into two separate categories. We have made edits to reflect this in revised figure 3 and the narrative text (see lines 297-363 of revised manuscript with track changes).

We reviewed the categories and themes under perceived barriers and think they are fine in the current form.

We reviewed the categories and themes under suggested solutions (as perceived by NTEP staff) and made some minor edits (lines 470-540 of revised manuscript with track changes) in the classification of themes under categories.

We have made edits here and there in the results narrative to improve clarity.

COMMENT
The discussion should highlight that ACF strategies need to be implemented considering the ground situations which is often lacking in Indian context. Capacity building and quality improvement are very general suggestions as these are constant processes. In my opinion authors need to provide suggestions based on their own specific findings which will help to undertake focused interventions.

RESPONSE

We thank the reviewer for the comment. We have added that ACF should be conducted considering the local context (lines 619 and 670 of revised manuscript with track changes). In addition, when there are huge and major knowledge and know-do gaps, capacity building must be done, in line with these ‘know-do’ gaps. Quality control linked incentives is a very specific suggestions we have made that is missing in the existing ACF guidance as well. Incentives should continue and they should be quality control linked. We have clarified this in detail in the discussion section (see lines 623-37 of revised manuscript with track changes) and have a separate figure for this as well as to what steps to follow to ensure quality control linked incentives (see Fig 5). While providing the explanation for this, we also provide the importance of use of ACF tool in the field for individual level data collection of those screened. ACF tool used in the field aids not only in ensuring provision quality control linked incentives, but also in regular implementation monitoring, and cross checking of aggregate numbers filled in Ni-kshay (lines 556-62 of revised manuscript with track changes). We have also provided specific suggestions as to how Ni-kshay ACF module should be simplified (lines 642-51 of revised manuscript with track changes).

COMMENT
Lines 472-473: “In line with our findings, studies elsewhere have also reported similar challenges [22–26]. Authors should first describe what other studies have reported and then discuss their findings whether they are confirming or having different observations.

RESPONSE

We thank the reviewer for the comment. We have made edits in this paragraph by first clarifying the barriers.

COMMENT
The conclusion needs to be specific and based on findings rather than general.

RESPONSE

We thank the reviewer for the comment. We have made the necessary changes. Please refer to lines 689-95 of revised manuscript with track changes.
